# Accelerating the Development and Validation of Liquid Biopsy for Early Cancer Screening and Treatment Tailoring

**DOI:** 10.3390/healthcare10091714

**Published:** 2022-09-07

**Authors:** Denis Horgan, Tanja Čufer, Francesco Gatto, Iwona Lugowska, Donatella Verbanac, Ângela Carvalho, Jonathan A. Lal, Marta Kozaric, Sinead Toomey, Hristo Y. Ivanov, John Longshore, Umberto Malapelle, Samantha Hasenleithner, Paul Hofman, Catherine Alix-Panabières

**Affiliations:** 1 European Alliance for Personalised Medicine, 1040 Brussels, Belgium; 2 Department of Molecular and Cellular Engineering, Jacob Institute of Biotechnology and Bioengineering, Faculty of Engineering and Technology, Sam Higginbottom University of Agriculture, Technology and Sciences, Prayagraj 211007, India; 3 Medical Faculty, University of Ljubljana, 1000 Ljubljana, Slovenia; 4 Department of Oncology-Pathology, Karolinska Institute, 171 64 Stockholm, Sweden; 5 Department of Soft Tissue/Bone Sarcoma and Melanoma, Maria Sklodowska-Curie National Research Institute and Oncology Centre (MSCI), 02781 Warsaw, Poland; 6 Department of Medical Biochemistry and Hematology, Faculty of Pharmacy and Biochemistry, University of Zagreb, Ante Kovačića 1, 10000 Zagreb, Croatia; 7 i3S—nstituto de Investigação e Inovação em Saúde, Universidade do Porto, Rua Alfredo Allen 208, 4200-135 Porto, Portugal; 8 INEB—Instituto de Engenharia Biomédica, Universidade do Porto, Rua Alfredo Allen 208, 4200-135 Porto, Portugal; 9 Institute for Public Health Genomics, Department of Genetics and Cell Biology, GROW School of Oncology and Developmental Biology, Faculty of Health, Medicine and Life Sciences, Maastricht University, 6211 LK Maastricht, The Netherlands; 10 Department of Molecular Medicine, RCSI University of Medicine and Health Sciences, Beaumont Hospital, Smurfit Building, D09 Dublin, Ireland; 11 Department of Paediatric and Medical Genetics, Medical University, 4000 Plovdiv, Bulgaria; 12 Astra Zeneca, 1800 Concord Pike, Wilmington, DE 19803, USA; 13 Department of Public Health, University of Naples Federico II, 80137 Naples, Italy; 14 Institute of Human Genetics, Diagnostic and Research Center for Molecular BioMedicine, Medical University of Graz, 8036 Graz, Austria; 15 Laboratory of Clinical and Experimental Pathology, FHU OncoAge, Pasteur Hospital, University Côte d’Azur, CEDEX 01, 06001 Nice, France; 16 Laboratory of Rare Human Circulating Cells (LCCRH), University Medical Centre of Montpellier, 641 Avenue du Doyen Gaston Giraud, CEDEX 5, 34093 Montpellier, France

**Keywords:** liquid biopsy, cancer, personalized medicine, technology, healthcare, policy framework, legislation, diagnosis, treatment, governance, implementation

## Abstract

Liquid biopsy (LB) is a minimally invasive method which aims to detect circulating tumor-derived components in body fluids. It provides an alternative to current cancer screening methods that use tissue biopsies for the confirmation of diagnosis. This paper attempts to determine how far the regulatory, policy, and governance framework provide support to LB implementation into healthcare systems and how the situation can be improved. For that reason, the European Alliance for Personalised Medicine (EAPM) organized series of expert panels including different key stakeholders to identify different steps, challenges, and opportunities that need to be taken to effectively implement LB technology at the country level across Europe. To accomplish a change of patient care with an LB approach, it is required to establish collaboration between multiple stakeholders, including payers, policymakers, the medical and scientific community, and patient organizations, both at the national and international level. Regulators, pharma companies, and payers could have a major impact in their own domain. Linking national efforts to EU efforts and vice versa could help in implementation of LB across Europe, while patients, scientists, physicians, and kit manufacturers can generate a pull by undertaking more research into biomarkers.

## 1. Introduction

The revolutionary precision that molecular diagnostics can bring to healthcare and the dramatic improvements it can bring to oncology screening and treatment are now widely recognized [1]. However, progress is hampered by several logistical and technical factors. Current molecular diagnostics is based on resected tissue samples, fine-needle aspirates, and needle biopsies [2,3]. Still, a majority of molecular diagnostics in tumors, such as those in the lung, are weighted toward needle biopsies [4]. The small amount of tissue obtained by needle biopsies may not capture the most aggressive subclones present, since individual tumors consist of diverse subpopulations (minor key clones can be easily missed). Moreover, some tumor entities, such as lung cancer, are located at remote sites, and a needle biopsy can be very difficult and constitute a high risk to the patient [5]. The mere analysis of the resected primary tumor alone (current standard practice in oncology) may provide misleading information with regard to the characteristics of metastases, the key target for systemic anticancer therapy. There are medical risks —particularly in children—from repeated anesthesia to obtain sufficient diagnostic and prognostic information [6]. Additionally, the biopsy of metastases is an invasive and sometimes dangerous procedure [7]. The promise of precision medicine as a model to customize healthcare to the individual patient, deploying new genetic tools to classify and characterize diseases and their hosts, has thus not reached its full potential [8]. To accomplish its goals relying only on traditional tumor sampling through needle and surgical biopsy, a massive increase in invasive procedures would be necessary to obtain sufficient material to accurately capture and describe genomic variations and their phenotypes, with obvious negative implications for resources and patient comfort [9,10]. Liquid biopsy (LB) offers an attractive alternative. It permits the sampling and analysis of non-solid tissue, primarily blood, as a screening, diagnostic, and monitoring tool for cancer [11,12]. Furthermore, as LB requires only a blood draw, it is a non-invasive procedure and constitutes a potentially more rapid and less costly alternative to genomic analysis of tissue biopsies [13]. More importantly, LB can be repeated during cancer treatment to monitor drug efficacy or to follow minimal residual cancer [14]. However, its predictive value and clinical utility are still to be compellingly demonstrated in Europe [15] and many of the related issues that must be better understood—in regard to cost and reimbursement, infrastructure, and skills—are also still under study. These aspects are moving faster in the US, with two FDA approvals and some encouraging trial results (see below). Progress on these questions and the introduction of LB methods and next-generation sequencing (NGS) technology for routine clinical practice should contribute to the overall improvement and personalization of anticancer therapy [16,17,18]. 

The European Alliance for Personalised Medicine (EAPM) decided to generate this work as an attempt to address these open challenges and to identify steps that need to be taken to effectively implement LB at the country level in Europe. EAPM-led expert panels were organized in the first half of 2022, including key stakeholders from across several European countries, covering medical, economic, patient, industry, and governmental expertise. Discussions focused on three key areas: early detection of cancer, i.e., the use of blood tests for screening and early detection of (multiple) cancers; the use of LB following curative treatment to guide adjuvant therapy; and cancer treatment selection and monitoring, i.e., the use of LB to select targeted treatment and monitor progressive disease and response to treatment. The aim was to determine how best to promote LB use in routine clinical care and to tackle the challenges this poses. The demand for LB tests is inevitably influenced by organizational issues relating to standardization, guidelines, and awareness among physicians and in the patient community, while supply of the tests is a function of the related infrastructure, arrangements for paying for procedures and materials, and how far underlying evidence generates calls for testing. Joint work at both the national and European level should concentrate on promoting cooperation among the widest range of stakeholders, the panels agreed.

## 2. State of Play

An LB is a simple and non-invasive method that emerged a decade ago as an attractive alternative and represents one of the most active research areas in oncology [14,19]. Circulating tumor cells (CTCs) are of utmost importance, as you can analyze the genome, transcriptome, proteome and the secretome in real-time, in addition to assessing the functionality of the most aggressive and disseminating clones. Circulating tumor DNA (ctDNA) enables real-time assessment of the tumor mutational profile and has therefore rapidly gained attraction [20,21]. The concept of LB was described and coined for the first time in 2010 by Drs. Alix-Panabières and Pantel [19] and initially described circulating tumor cells in the peripheral blood. Nowadays, the definition has been expanded to include all circulating tumor-derived biomarkers (e.g., circulating tumor DNA; and extracellular vesicles, such as exosomes, microRNA, tumor-educated platelets, etc.), as well as immune cells in all body fluids (e.g., bone marrow, urine, and sputum) [14]. These biomarkers offer complementary information at different levels. Numerous LB-based studies and clinical trials for a wide variety of cancer types (e.g., breast cancer, colorectal cancer (CRC), prostate cancer, non-small cell lung cancer (NSCLC), and malignant melanoma) have been initiated to demonstrate their clinical relevance in cancer patients [14,22,23]. The clinical utility of LB has been demonstrated for the detection of epidermal growth factor receptor (EGFR) mutations in NSCLC patients or for the detection of KRAS proto-oncogene, GTPase (KRAS) mutations in patients suffering from metastatic CRC [24]. When comparing patients’ outcomes on targeted therapy based on LB and tissue, similar results have been revealed for NSCLC patients [25,26,27]. If the tissue-based biopsies are not available, if they are low quality, or if they involve significant risk to obtain them, guidelines on national and international level now include companion diagnostic tests as an alternative [22]. For companion diagnostics for several cancer types, the clinical utility of LB has been proved. Moreover, four companion diagnostic tests have been approved to date. They are the cobas EGFR Mutation Test v2 from Roche (“a quantitative PCR (qPCR)-based test for the detection of EGFR exon 19 deletions, the NP_005219.2:p.L858R substitution in metastatic NSCLC patients to identify eligibility for TKI treatment, as well as for the EGFR NP_005219.2:p.T790M resistance mutation”) [22,28]. The “Guardant360^®^ CDx from Guardant Health to determine EGFR status in NSCLC patients” and the “FoundationOne^®^ Liquid CDx from Foundation medicine for NSCLC, metastatic castrate resistant prostate cancer (mCRPC), ovarian and breast cancer patients before administration of TKI, PIK3CA, or poly (ADP-ribose) polymerase 1 (PARP) inhibitors” are two NGS-based tests that were recently approved by FDA. G360 CDx and F1L CDx, in addition to the FDA-approved CDx claims, include the majority of actionable targets on their panels, and these have been analytically validated. These providers deliver not only the CDx claims for the approved therapies but also comprehensive reports with molecularly guided treatment and trial options which are also actionable in many settings especially in the US. “Epi proColon^®^ (Epigenomics AG, Berlin, Germany)” is a blood-based test which is, for now, the only test that detects tumor-associated epigenetic changes and is FDA-approved for CRC screening [22,29]. These US approvals in effect offer recognition of clinical utility and US studies are revealing unexplored potential in plasma-based NGS performed simultaneously with diagnostic biopsy in suspected advanced NSCLC [30], while other studies suggest that LB has emerged as a viable approach to guide therapeutic decisions and provide real-time follow-up in NSCLC [31]. 

These optimistic findings are echoed in the European Society for Medical Oncology (ESMO) paper summarizing international studies of scenarios where ctDNA mutation testing may be implemented in clinical practice, highlighting where ctDNA LB may be considered as a complementary tool to TB analysis to provide the full picture of patients’ actual predictive profiles, as well as ctDNA mutation testing to assist when a patient has a discordant clinical history and is suspected of showing intertumor or intratumor heterogeneity [32,33]. A Canadian study found that incorporation of TL-LBx-CGP demonstrated an overall impact of CAD 14.7 million with 168 life-years gained in the publicly funded healthcare system in the 3-year time horizon [34]. A consensus statement from The International Association for the Study of Lung Cancer (IASLC) concludes that LB represents a practical alternative source for investigating tumor-derived alterations [35]. However, another ESMO paper is more cautious, suggesting that the clinical utility of an early diagnosis of progression has not yet been demonstrated in randomized clinical trials with adequate cohorts of patients [15]. 

The development of LB assays is often based on the analysis of whole-genome sequencing (WGS) or whole-exome sequencing (WES) data from tumor tissue samples. It can be further processed into large and/or customized gene panels. The Integrated Mutation Profiling of Actionable Cancer Targets gene panel is FDA approved and was originally developed as a next-generation sequencing (NGS) hybrid assay for targeted deep sequencing. The FDA has already approved several single-gene tests and, more recently, multigene tests to detect genetic changes in plasma cell-free DNA (cfDNA). They would be used as companion diagnostics aligned with specific molecularly targeted therapies for cancer. These approvals are a major milestone for the widespread use of LB in the clinic, particularly in patients with advanced cancer. Currently, cfDNA tests are only approved as a companion diagnostic for a few specific types of cancer and targeted therapies. Outside of these indications, it would be necessary to screen many patients to identify the small percentage of patients who could benefit from drugs approved by a regulatory agency [36]. Whether these initial assessments will last depends on progress in translating findings from prospective clinical trials and real-world evidence databases into new indications for new or existing molecularly targeted therapies [37].

Cancer screening and Diagnosis: Potential future applications

The researchers have, in addition to the tissue-based MSK-IMPACT test, developed the MSK-ACCESS (Cell-Free Analysis of Circulating DNA for Assessment of Somatic Status) test. It uses deep sequencing of plasma cfDNA for broad coverage of cancer-related genes. This LB test was approved in 2019 by the New York State Department for use in the identification of molecular and cellular tumor markers. Some of the key aspects that must be considered during test development are the type of sample analyzed, specific sample-collection procedures, processing, handling, and storage, as well as specific characteristics related to the patient. The standardization of pre-analytical variables is considered an important part of the test development process. For example, the ESMO Scale of Actionability of Molecular Targets is one example of an attempt to facilitate this process by ranking genomic aberrations according to their importance for precision medicine [38]. Clinical adoption of the LB test should be achieved through three distinct steps. The first step involves test development and validation; the second is regulatory approval, which means inclusion in guidelines and reimbursement; and the third is inclusion in the clinical workflow [37].

LB assays in advanced-stage disease

LB assays can be used in patients with advanced stage cancer when choosing the right treatment to monitor the effectiveness of the treatment and to determine the most appropriate follow-up treatment if resistance develops [37].

The right treatment for the right patient

PCR-based cfDNA assays for oncogenic driver variants of genes such as EGFR in non-small cell lung cancer (NSCLC) and KRAS in colorectal cancer (CRC) showed high specificity (mean 96%) but only moderate sensitivity (mean 66%) when compared with tumor tissue assays, which are still the gold standard [39]. Nevertheless, the use of droplet digital PCR (ddPCR) assays increases the level of sensitivity. It should be noted that only few data are available on the effects of patient-related factors such as pregnancy, smoking, exercise, and various non-malignant conditions that might affect cfDNA levels in blood. Thus, the correlation between patient-related factors and performance of specific ctDNA assays should be carefully explored in prospective studies. The development of collection tubes for long-term storage of viable CTCs is an important unmet need for the evaluation of the full potential of these analytes in multicenter trials. Moreover, the typically low numbers of CTCs present in blood samples also hamper testing of the clinical utility of CTC-based functional assays or omics analyses [40].

Detection of resistance mechanisms

The frequent occurrence of co-mutations or copy-number alterations (CNAs) and the development of resistance mutations is often a major challenge in the application of ctDNA in guiding treatment decisions. With the use of plasma ctDNA analysis, different mechanisms of resistance to targeted therapy can be monitored; this also includes co-mutations that can affect treatment decisions in multiple cancer types, most notably in patients with NSCLC96 and CRC [41]. Besides monitoring for known resistance mechanisms, cfDNA can be used to identify unknown mechanisms of treatment resistance. One of the first studies in this area that involved the analysis of serial plasma cfDNA samples by using WES provided insights into the mechanisms of resistance to commonly used chemotherapeutic or targeted agents. A patient with metastatic HR+/HER2− breast cancer, e.g., who progressed following paclitaxel treatment had increased mutant allele fractions of PIK3CA, BMI1, and SMC4 [42].

“ctDNA relapse”

“ctDNA relapse” is a relatively new term describing a “disease stage in which patients present with detectable ctDNA during routine cancer surveillance but without overt imaging-detected disease relapse after completion of surgery and neoadjuvant and/or adjuvant chemotherapy for their primary cancer. Systemic treatment for ctDNA relapse has the potential to create a drug-testing setting that goes beyond metastatic, adjuvant, neoadjuvant, and post-neoadjuvant settings that have been traditionally used in clinical trials [37].

Liquid biopsy for early cancer detection

In addition to the challenges associated with low levels of ctDNA in early stage cancer, the low incidence of cancer in the general population is also a significant challenge for the use of LB [37]. Although the key disadvantage of CTCs and ctDNA as biomarkers is that they are undetectable in many patients with early stage and some with advanced-stage cancer, several LB approaches for cancer diagnosis are under development [37]. Initial approaches have been based on the detection of driver gene mutations in plasma cfDNA [43]. Another approach is based on combined analyses of circulating proteins and cancer-associated mutations in plasma, such as the CancerSeek platform [44]. Other approaches are predicated on the analysis of epigenetic alterations that might be tissue-specific and cancer-type-specific by analyzing genome-wide differentially methylated regions via cell-free methylated DNA immunoprecipitation and high-throughput bisulfite-free sequencing (cfMeDIP-seq)165 or other methylation patterns [45,46]. An example of the potential clinical utility of an LB approach for early detection has been provided by the DETECT-A study [47]. In this prospective study, 10,006 women aged 65–75 years with no prior history of cancer were evaluated by using the CancerSeek platform. The study showed that 26 women had cancers that were detected by using this platform. Five of them with stage I (19%), three with stage II (12%), eight with stage III (31%), and nine with stage IV (35%) cancers, as well as one with cancer of unknown stage but without metastases [47].

Psychological aspect

Another dimension that must be taken into account is the psychological aspect of this type of technology. The traditional meaning of what it means to be “a patient” or “to have a disease” can notably be changed with the availability of LB assays for the early detection of cancer. Although for some otherwise healthy, asymptomatic people, the fact that the disease is identified at an early stage will be an important discovery, for many, the diagnosis could be unwanted and potentially destructive [37].

Artificial intelligence (AI) and LB

Artificial intelligence has great promise to revolutionize the way medicine is practiced. It has already been leveraged to improve the performance of different LB assays, and this will also facilitate their further integration into the clinical workflow. Some examples are the use of machine-learning approaches for the detection and characterization of CTCs; for the analysis of ctDNA for cancer detection and localization; and for integrative multi-omics analyses and future integration of LB tests together with other clinicogenomic, metabolomic, immunomic, microbiomic, and homeostatic data to guide treatment decisions [48,49]. A machine-learning platform named “lung cancer likelihood in plasma” (Lung-CLiP) is being developed for early lung cancer detection based on targeted sequencing of plasma cfDNA and matched leukocyte DNA [37].

### 2.1. National Perspectives

Different perspectives are observed in various European countries regarding the implementation and current status of LB in healthcare systems (Table 1).

In Austria, recent translational research has focused on liquid biopsies to guide precision medicine, with plasma increasingly being used for molecular profiling to guide treatment decisions for patients with solid tumors and progressive disease [50]. At several academic hospitals, there is reimbursement of comprehensive genomic profiling (CGP) of both tumor tissue and liquid for select patients with progressive disease. However, doctors disagree on which strategy is most appropriate—a nationwide program involving community radiologists, or screening programs in well-established cancer centers with all the required infrastructure already in place [51]. At the Institute of Human Genetics at the Medical University of Graz, one of the leaders in LB research in Austria, novel multiparameter bioinformatics approaches are being developed in several translational projects for patients with breast, colorectal, and prostate cancer for early detection and monitoring purposes. At the same university, funding from the FWF Top Citizen Science initiative was received to educate patients with breast cancer and to identify novel precision oncology approaches that help them better understand how treatment decisions were made based on molecular profiling via LB. Similarly, a platform was recently launched to educate and guide oncologists in the implementation of LB in the clinic, as well as to create transparency in complex ctDNA strategies with future application, such as nucleosome mapping and fragmentomics [52].

Bulgaria’s Center for Competence was involved in a Personalized Innovative Medicine PERIMED project that was co-funded by the EU and the Science and Education for Smart Growth Operational Programme. However, there is little or no coverage of tests by national insurance, usually leaving patients to pay for the tests [53]. 

Croatia’s national cancer control plan envisages implementation of new, validated, and cost-effective cancer molecular testing procedures with the aim of applying targeted oncological therapies and includes LB for genetic, protein, and RNA profiling in early CRC diagnosis. There is strong support for the concept of better collaboration in Europe [54].

LB is not currently routinely used in clinical practice in Ireland. Standardized methods will need to be in place before widespread use, particularly for adjuvant treatment decisions. Integration into cancer care will present organizational and financial challenges. Provision of appropriate skilled staff requires additional investment. However, the authorities recognize the need for incorporation of advances in diagnostics into clinical care pathways and clinical trials show that LB analysis is feasible, cost effective, and acceptable to patients.

In France, the STIC METABREAST clinical trial, a medico-economic trial, demonstrated for the first time the clinical utility of CTCs in metastatic HR + breast cancer patients [55]. The quality of life of these patients has also been evaluated. The High Authority of Health (HAS) is currently evaluating whether to refund this blood CTC test by French social security. It would be a great first step to introduce LB in clinical practice. Moreover, the IDEA-FRANCE trial concluded that plasma ctDNA testing opens up an opportunity for precision treatment of patients with localized CRC. The study is also one of the first to show that, in the future, it may be possible to use LB to direct therapy and identify which patients can avoid chemotherapy after their surgery and which should have it. LB units were developed in 1999 at the University Medical Center of Montpellier and in 2009 at the Laboratory of Clinical and Experimental Pathology, Pasteur Hospital, University Côte d’Azur. They initiate and lead translational research programs funded by the French NCI [56]. France uses LB at progression for targeted therapy, for clinical trials, and for research projects. NGS availability varies widely from city to city [57]. The complex reimbursement system impedes wide use, including issues of confusion over which technology—in-house or on commercially available platforms—should be used. There is now a program for the early detection of lung cancer by using LB in association with low-dose computed tomography (LDCT), as well as an artificial intelligence program supported by the National Cancer Institute and some pharmaceutical compagnies in a pioneering integration of different parameters.

In Germany, CRC patients insured by BARMER are covered for the OncoBEAM RAS CRC IVD Test, an innovative LB diagnostic that delivers a comprehensive evaluation of RAS mutations from a single tube of blood. The German genetic testing firm CeGaT has received funding from the German Federal Ministry of Education and Research KMU-Innovativ: Biotechnologie-BioChance programme to support the development of LB methods for the analysis of ctDNA and expand its genetic analysis services to new patients who would currently be ineligible because they cannot safely undergo a tissue biopsy [58]. 

In Italy, Cancer LOCATOR is a cfDNA-based method which uses CpG methylation profiles and thus allows for detection and predicts the tissue of origin [59]. In another study, cardiomyocyte death, which is a potentially useful marker, was identified by using cfDNA methylation profiles. The result of these studies was that these markers could potentially be used in clinical practice to predict or to diagnose certain clinical conditions [60].

In the Netherlands, the COIN-consortium is a nationwide initiative in which a team of multidisciplinary specialists are working toward a coordinated clinical implementation of LB ctDNA analysis as an innovative form of minimal invasive molecular diagnostics in clinical practice. These studies focus on colorectal and non-small cell lung cancer, as there is a substantial and active ctDNA research community for these two tumor types. Patients with stage II colon cancer will be offered adjuvant chemotherapy based on the presence of ctDNA in their blood after surgery [61].

The EU-funded Lima aims to develop and validate technologies and tools to include liquid biopsies in the clinical workflow, aiming at introducing a more precise and dynamic genetic characterization of tumor at the diagnosis and during treatment phases [62].

In Norway, the Norwegian Regional Research Program ran a five-year research program on LB entitled “Personalised cancer therapy—biomarkers in clinical trials”. The biggest challenge is the difficulty to standardize techniques across projects in the face of the continuous development of new technologies [61].

In Poland, current guidelines indicate that, in patients with progression during EGFR TKI treatment, it is important to re-sample material in order to evaluate the potentially acquired T790M mutation in the EGFR gene, as this is strongly associated with developed resistance to TKI. It is recommended to perform tests in cfDNA first, and only if these results are negative, re-biopsy or needle biopsy should be performed. There is strong support for standardization of an approach at the national and EU level in terms of diagnosis and in treatment [63].

In Slovenia, LB is currently used in everyday clinical practice for detecting T790M mutations in NSCLC patients progressing on first- or second-generation EGFR TKIs and for primary molecular diagnostics of advanced non-squamous NSCLC patients who are not candidates for tissue biopsy. In addition, there are ongoing studies on the use of LB to guide adjuvant therapy in solid tumors, and there is strong support for joint EU efforts in screening and for the use of LB in advanced screening for lung cancer [64].

Sweden provides strong support for innovation with grants and strong intellectual property rights and start-up support, but the healthcare market is regionalized, thus leading to disparate decision-making bodies for health technology assessment.

At the European level, CANCER-ID, a five-year Innovative Medicines Initiative (IMI) consortium, which came to an end in 2019, published best-practice protocols and the results of ring studies based on the implementation of harmonized protocols and standard materials [65]. Against this background, LB protocols are being explored in clinical studies. CTCs and ctDNA assays were evaluated for their predictive and clinical utility in the use of immune checkpoint inhibitors. Except bringing LB into clinical trials and practice, the project is also supporting LB research in a wide range of applications. The particular merit of the project is that it provides a platform for bringing together regulators, healthcare professionals, and patients with academia and industry. The impact of the work is being amplified by the drafting of guidelines, and training and communication strategies so as raise the profile of Europe in the field and boost international research collaborations (in the US, Asia and Australia) [66]. In a continuum of CANCER-ID, the novel EU consortium, the European Liquid Biopsy Society (ELBS), established by the University Medical Centre Hamburg-Eppendorf (Prof Klaus Pantel), aims to become the leading hub for LB research in Europe with the goal to translate LB assays into clinical practice for the benefit of patients [67,68].

Buyers in Belgium, France, Germany, Italy, and Spain cooperate in the oncNGS consortium, challenging the market to develop novel affordable solutions to provide the most advanced NGS tests for cancer patients. They wish to co-develop an EU innovative tender for LB diagnostics based on complex NGS-driven DNA profiling within a pre-commercial procurement Horizon-2020 financed project [69].

### 2.2. Opportunities

Plasma ctDNA analysis has opened previously unexpected perspectives for monitoring cancer genomics in the peripheral blood [70]. The use of LB as a form of early diagnosis and screening for cancer patients has emerged as an attractive option, offering multiple benefits in patients with diverse tumor types with a highly precise evaluation of the tumor genomic alteration landscape, reflective of disease burden. In addition to the role that it could play in more sensitive and specific screening, LB has enormous potential as personalized medicine increasingly expands into the clinic and more therapeutics are actively being developed and used in patients based on the molecular profiles of their different cancer types [2,71]. The discovery and classification of these biomarkers is of utmost importance. Solid tumors are not static, but rather they are continuously growing, spreading, and changing their microenvironment, as well as shedding intact cells and cell components into surrounding body fluids. LB serves as a safe alternative to solid biopsies (complementary information is given by both biopsies) and is thus a useful and critical component to fully realizing personalized medicine. Their use extends beyond screening and selecting a targeted treatment for monitoring progressive disease and response to treatment [6,72]. LBs have been used in the field of immuno-oncology (I-O) to predict response, relapse, or adverse advents for patients undergoing immune-checkpoint inhibitor (ICI) therapy (anti-PD-1/PD-L1 and CTLA-4). Alongside the quantification of cfDNA as a predictive biomarker, there is additionally the quantification of PD-L1 from CTCs that are bound on exosomes or free in plasma, as well as the determination of cytokines, as it is being actively investigated with promising results [73].

LB is an important milestone in the modernization of oncology care, granting access to critical information for timely selection of the most appropriate therapy. The technology offers genetic analysis services to new patients who would currently be ineligible because they cannot safely undergo a tissue biopsy. In addition to the primary diagnosis, the analysis of CTCs and DNA also allow close for the monitoring of cancer in ways that tissue analysis does not [74,75,76].

The main goals in determining treatment are to choose the most effective course for an individual patient, targeting the subpopulation of patients who will benefit most from a particular medicine and avoiding toxic therapies for patients who do not need them [77,78]. Plasma ctDNA testing and CTC enumeration plus phenotyping open up great opportunities for precision treatment of patients with localized and metastatic solid cancers [74,79]. 

In addition, this type of research has an important role in public health because of its ability to provide more efficient cost control. The advantages of LB include high-throughput assays, with minimal sample, reagents, and waste production. LB has health economic potential if used to initiate and serially monitor treatment response, to inform decisions to discontinue inactive treatment, or to switch treatment to agents that target other molecular mechanisms in case of resistance [75]. In the future, it may be possible to use LB to direct therapy and identify which patients can avoid chemotherapy after their surgery and which should have it. CTC/ctDNA for minimal residual disease (MRD) is strongly prognostic in early cancers, and LB is a perfect tool for MRD detection that may allow for the personalization of adjuvant/consolidation therapy [80].

Some key advantages are better accuracy in clinical settings, tertiary prevention with MRD after treatment for the early stage, monitoring the disease burden and emergence of resistance in the metastatic setting, and identification of actionable alterations for targeted therapy in trials or approved medicines [81].

In lung cancer, with its high mortality; disease heterogeneity, pattern of late diagnosis; the limitations of tissue biopsy (and re-biopsy), particularly because of tumor location; and the challenge of intra-tumoral heterogeneity, LB offers new hope [82]. LB for biomarker testing in NSCLC confers advantages. In Europe, tissue biopsy may be unfeasible or inadequate for molecular workup in an estimated 16,000 patients annually with NSCLC; these patients could potentially benefit from LB. Based on published frequencies of NSCLC driver alterations, a molecular diagnosis based on LB could allow approximately 6560 patients annually to benefit from current, emerging, and future targeted treatments [83,84,85]. A study in 2022 investigated whether a ctDNA-guided approach as compared with a standard approach in stage II colon cancer can reduce the use of adjuvant treatment without compromising the risk of recurrence. The results showed that a ctDNA-guided approach reduced adjuvant chemotherapy use without compromising recurrence-free survival. Among ctDNA-negative patients, 3-year recurrence-free survival was higher among patients with clinical low-risk cancers than among those with high-risk cancers. Nevertheless, further studies and data will be needed to finalize and prove all the results [86].

### 2.3. Challenges

To successfully implement LB into standard care, certain barriers have to be overcome. Besides considering technical issues related to acquiring proper workflows, the quality of LB testing should also be assured. The comparability of LB test results is also very important part since it is a prerequisite for reliable diagnostics and reimbursement.

Some of the technical issues related to LB are as follows:

- The low concentration of the circulating biomarkers at early stage of cancer; a solution is (i) to increase the blood volume and (ii) to combine different circulating biomarkers to be more sensitive;
- For ctDNA: possibility of fragmentation, the low fraction of ctDNA in total amount of cfDNA [22].

It is considered important to standardize pre-analytical and analytical procedures to ensure reproducibility and generate structured and accessible clinical reports. Pre-analytical variability is a vital issue. Errors in the pre-analytical phase—specimen collection and processing, transport and storage, cDNA isolation, and quality controls—can heavily affect the data generated in the following analytical steps, resulting in unreliable results which ultimately can lead to incorrect clinical decisions [87].

The quality assurance of LB in terms of internal and external quality control (QC) is critical to ensure reliable test results. The internal QC of the pre-analytical workflow should assess cfDNA yield and integrity. Appropriate external controls should be analyzed in parallel, as well, to evaluate ctDNA analysis [22,88].

The clinical validity of LB (measured as the capacity of a test to divide a population into groups with significantly different clinical results) and the clinical utility (measured as the capacity of a test to improve cancer diagnosis, treatment, management, or prevention results) are the objectives of current oncology studies on LB. More interventional clinical trials must be initiated to demonstrate the clinical utility of LB and to introduce this test in clinical practice [67]. To date, LB is not considered a sufficiently sensitive or specific technique for early cancer detection in an asymptomatic population and cannot substitute for or complement radiological tests [89].

Multidisciplinary molecular tumor boards are needed to oversee these processes and to enable the most suitable therapeutic decisions for each patient according to the genomic profile [89]. The way that the costs of genomic medicine are covered strongly influences access to testing. Both directly and indirectly, the arrangements for payment have an impact on which patients benefit, on whether they then received targeted therapies, and, consequently, on the outcome of their care [90].

The cost-effectiveness of LB is a requirement for adoption and reimbursement in many countries with a Health Technology Assessment (HTA) program. The benefits of LB have been positively evaluated in initial cost-effectiveness studies, and recent increases in private- and public-payer reimbursement for LB testing have been noted [22]. It is still not widely adopted in Europe, and reimbursement options are limited to a few applications in only several countries. Furthermore, recognition by health insurers is a lengthy process, so there is an urgent need for prospective large-scale clinical trials for promising LB applications. In Europe, a shift by public hospitals toward greater use of LB in NSCLC—where tissue biopsy still predominates—is conditioned by regional policies [85], with some moves toward paying for EGFR mutation testing (in Italy, Slovenia, and the UK, for instance), but with widespread hesitation over other biomarkers. In the study by Wu et al. [91] it was pointed out that LB use is frequently restricted to monitoring the resistance to EGFR TKIs, and more recent studies list LB assays approved in Europe still limited to *EGFR* mutation testing [92]. The uptake of NGS-based LB faces even greater obstacles in Europe since NGS is not reimbursed in many of its countries [57].

Highly specific tests are needed for screening purposes. Low prevalence will strongly increase false positives and decrease positive predictive value. With 95% specificity and 95% sensitivity and a prevalence of 1:2000, 10,000 tests will generate 500 false positives and 5 true positives, providing a PPV of less than 1% [93]. Every false positive will generate extra costs of further testing, anxiety, and potential burden of invasive or radiology testing. Integration of LB assay into clinical practice could bring advantages of ease and accuracy. There is a need for more sensitive and specific testing [94].

### 2.4. Identified Issues and Challenges from the Panels

The anecdotal evidence from the EAPM panels included the following areas of weakness in the current arrangements:

- Failings in communication among HCPs: A considerable number of pathologists and clinicians especially are not yet familiar with NGS results and with LB outside of a clinical trial or a research study, so they receive a report and do not know what to do with it;
- Information, guidance, and recommendations vary too much across Europe;
- Clinicians often take distinct approaches in response to the same results;
- Lack of molecular tumor boards in some parts of Europe;
- Patient awareness is insufficient, and patient contact is too limited: Patients who take part in research studies come in to get a blood draw and receive little or no feedback;
- Limitations remain regarding the use of LB and how it helps patients when we do not have treatments for ovarian cancer;
- Limited access to early stage samples of cancers;
- No universal standard for sample processing exists;
- Absence of networks for sending patients to clinical trials across borders;
- Lack of reimbursement deprives patients of access, but also discourages innovators from investing;
- Technology and methods are always evolving, making them overwhelming to incorporate.

### 2.5. Moving toward Solutions

Demonstrating the clinical utility of LB and its usefulness for research would be assisted if there were easy-to-use, robust, and reproducible workflows. Currently, there are no integrated multicenter-tested workflows available covering the requirements for the clinical setting. Such workflows should include Standardized Operating Procedures (SOPs) for all of the abovementioned phases of laboratory testing starting with specimen collection and ending with result interpretation, e.g., via bioinformatics analysis. An international LB standardization alliance is needed among organizations and foundations that recognize the importance of working toward the global use of LB in oncology practice to support clinical decision-making and regulatory considerations and seek to promote it in their communities [10].

LB can increase access to testing in advanced cancer, but it requires an awareness of profiling opportunities, an understanding of the methodologies and the results, conversion of the results into actionable insight, reimbursement strategies, and expert guidance for the interpretation and application of cfDNA analyses [52].

Overall, precision oncology demands polymath proficiency across multiple knowledge domains in order to decide which patients to test; which panels to order and for which indication and issue (for targeted therapy at baseline, for targeted therapy at progression, for immunotherapy at baseline, for prognosis purpose in MRD, or for screening and early detection); with which technology; and whether outsourced or in-house, which labs to use; how to standardize value-based precision medicine hospital-wide; and what the results mean. It demands the ability to answer questions from patients as to why they are receiving their particular treatment, and whether a particular test will improve their outcome. At the level of health systems, it needs to be determined how to drive volume and enhance reimbursement, as well as decide which tests should be reimbursed and for which treatments [11,95]. Precision oncology and information overload are a problem in that technology, evidence, and approvals are always changing. There is a pressing need for designing and executing adaptive clinical trials based on LB/MRD detection in the neoadjuvant/adjuvant setting to establish their clinical utility, along with improvements to CTC/ctDNA assays’ sensitivity and reproducibility [96].

### 2.6. EU Engagement

The EU Beating Cancer Plan, the Cancer Mission, the EHDS, and the evolving EU pharmaceutical legislation all offer scope for progressing LB through funding and other support. Notably, however, two of the four key objectives in the Cancer Mission are “prevention and early detection” and “diagnosis and treatment”.

The plan specifically mentions LB in its priorities: “the Mission will develop non-invasive (or minimally-invasive) cancer screening and detection methodologies (‘integrated diagnostics’—imaging, tissue, fluid, clinical biomarkers), also using Artificial Intelligence. In dialogue with the Member States and with support from the JRC, it will bring them into everyday medical practice with the aim to enhance participation of the target population in population-based screening programmes”. There will be a role for academia, citizens/patients, SME/industry in the implementation, and the subject is a priority for funding in the Horizon Europe work program. The mission will “support an innovative clinical trial program focused on diagnosis optimisation, building on existing and minimally invasive diagnostic techniques, including imaging, and/or implementation research of validated AI powered integrated diagnostic methods (e.g., imaging, tissue, fluid, clinical biomarkers)”, with academia, citizens/patients, and SME/industry/charities, healthcare providers foreseen as involved in implementation. It also aims to improve the performance of the existing screening programs to make them faster, more accurate, and more personalized, as well as, through research, develop new screening tools that can be integrated in new screening programs and easily implemented at national level. There is an explicit role for academia, Member States, regions, healthcare, and insurance providers in the plan [97].

## 3. Recommendations

The recommendations (Table 2) emerging from the roundtable panels fall into three main categories: harmonizing the current inconsistencies at national and European level, improving the management and organization of the work of those involved in LB, and outreach to relevant stakeholders to improve awareness and to influence the policy environment.

## 4. Discussion

If LB is to have an impact on care, it has to be used in clinical practice, and the challenges are likely to be best resolved if there is closer collaboration both at the EU and at the national level among payers, politicians, healthcare professionals, and the research community—along with, of course, input from patient organizations. The current limitations notably include the need for more clinical trials to assess what could be the clinical importance of detection of tumor heterogeneity with ctDNA testing. Regarding other areas of implementation of ctDNA, such as screening, MRD assessment, etc., the evidence is lacking to provide proper recommendations. New technologies are under development that have the chance to provide the evidence for decision-making in clinical settings for the adoption of ctDNA assays [98].

Pharma companies, regulators, and payers around the EU, as well as at the global level, could conduct studies in which the LB serves as the patient selector (prognostic) or as treatment decider (predictive). Regulators could endorse LB-based endpoints, as preliminary approval and payers could use them as starting points for risk-based reimbursement discussions, and they could also generate real momentum as a push factor. Implementation of LB across Europe can be aided by linking national efforts, where care is delivered to in-need patients, with those at the European Union level. Furthermore, patients, scientists, physicians, and kit manufacturers can generate a pull by highlighting the benefits of convenience, undertaking more research into biomarkers, using LB more (if needed on research budgets), manufacturing cheap but mostly reliable kits, and financing QA ring schemes

The current wave of EU health-related legislation and policies—and, notably, the Beating Cancer Plan—provide a conducive background for focusing attention on the potential of LB. The EU Beating Cancer Plan presented in February 2021 takes new technologies, research, and innovation as its starting point and sets out a new EU approach to cancer prevention, treatment, and care, tackling the entire disease pathway, from prevention to the quality of life of cancer patients and survivors. Among its flagship actions are the creation of a Knowledge Centre on Cancer to better coordinate scientific and technical related initiatives at the EU level, an initiative on Cancer Diagnostic and Treatment for All to provide access to innovative cancer diagnosis and treatments across 2021–2025, and a EU Cancer Screening Scheme that will update recommendations on screening and new guidelines and Quality Assurance schemes over 2022–2025. The parallel EU Cancer Mission is bringing together Member State actions on understanding cancer, prevention and early detection, and diagnosis and treatment. The scope for the emergence of a conducive policy environment is also enhanced by concurrent legislation to update the EU In Vitro Diagnostic Regulation and by the prospect—from late 2022—of a wide-ranging review of EU pharmaceutical legislation, which has, as one of its main objectives, the need incorporate innovation into healthcare. The European level is also important in the discussion, since joint work there can help avoid the complications that could arise if individual national approaches are unaligned and consequently lead to fragmentation rather than cooperation.

## 5. Conclusions

The well-documented deficiencies of the current screening and diagnostic techniques hold back the fuller exploitation of molecular diagnostics and stand in the way of the evolution of personalized medicine. Somewhat perversely, the demonstrable attractions of LB continue to be underappreciated. Although evidence of its predictive value in clinical utility is still evolving, enough is already known about its potential to justify further exploration. Experience is showing its value in monitoring treatment response and prognosis, as well as in cost-effectiveness.

In 2022, ESMO published recommendations about ctDNA as a part of genomic testing and its potential use in clinics. Many different aspects of ctDNA have been reviewed by the ESMO Precision Medicine Working Group, which gave some perspective as to what the future of ctDNA testing could be. The recommendations and conclusions include taking incomplete sensitivity into account into clinical use—in particular, lower sensitivity for gene fusions and copy number events—and developing tests to differentiate correct results for further advanced genotyping [98].

The EU’s 2022 plan to extend its 20-year-old guidance on cancer screening to a wider range of tumor types—something that EAPM has advocated since 2017—offers some additional hope of recognition of LB. Lung and prostate cancer are likely to be added. Moreover, as the EU plan itself suggests, improvements in screening and early diagnosis will require updates on the practical tools, as well as policy pronouncements. The plan notes the need for incorporating wider use of more recent technologies, such as digital breast tomosynthesis in breast cancer and HPV testing in cervical cancer. No mention is made of LB, but for full effect, updating recommendations should reflect the full potential of screening technology, which has moved fast and far since 2000. The EU should encourage the development of LB alongside other innovative methods by explicitly including it in its updated recommendation. Together with continued technological and scientific advances bringing greater precision and predictability to LB, there is a role for policy in enhancing the environment for further development.

## Figures and Tables

**Table 1:** Different perspectives and challenges among European countries with implementation of LB in the healthcare system.

Country	Perspective
Austria	- Novel multiparameter bioinformatics approaches are being developed for patients with breast, colorectal, and prostate cancer - A platform was launched to educate and guide oncologists in the implementation of LB in the clinic
Bulgaria	- Center for Competence was involved in a Personalized Innovative Medicine PERIMED project - Little or no coverage of tests by national insurance
Croatia	- National cancer control plan envisages implementation of new, validated, and cost-effective cancer molecular-testing procedures - Aim is to apply targeted oncological therapies and include LB for genetic, protein, and RNA profiling in early CRC diagnosis
Ireland	- LB is not currently routinely used in clinical practice - Clinical trials show LB analysis is feasible, cost effective, and acceptable to patients
France	- STIC METABREAST clinical trial demonstrated for the first time the clinical utility of CTCs in metastatic HR (+) breast cancer patients - IDEA-FRANCE trial concluded that plasma ctDNA testing opens up an opportunity for precision treatment of patients with localized CRC
Germany	- CRC patients insured by BARMER are covered for the OncoBEAM RAS CRC IVD Test - German genetic testing firm CeGaT has received funding to support the development of LB methods for the analysis of ctDNA
Italy	- Cancer LOCATOR is a cfDNA-based method which uses CpG methylation profiles and thus allows for the detection and prediction of the tissue of origin - Cardiomyocyte death, a potentially useful marker, was identified by using cfDNA methylation profiles
Netherlands	- In the COIN-consortium a team of multidisciplinary specialists are working toward a coordinated clinical implementation of LB ctDNA analysis - Patients with stage II colon cancer will be offered adjuvant chemotherapy based on the presence of ctDNA in their blood after surgery
Norway	- Norwegian Regional Research Program ran a five-year research program on LB entitled “Personalised cancer therapy–biomarkers in clinical trials” - The biggest challenge is the difficulty to standardize techniques across projects
Poland	- Current guidelines indicate that, in patients with progression during EGFR TKI treatment, it is important to re-sample material in order to evaluate the potentially acquired T790M mutation in the EGFR gene - It is recommended to perform tests in cfDNA first
Slovenia	- LB is currently used in everyday clinical practice for detecting T790M mutations in NSCLC patients progressing on first- or second-generation EGFR TKIs and for primary molecular diagnostics of advanced non-squamous NSCLC patients who are not candidates for tissue biopsy - Ongoing studies on the use of LB to guide adjuvant therapy in solid tumors
Sweden	- Provides strong support for innovation with grants and strong intellectual property rights and start-up support - Healthcare market is regionalized, which leads to disparate decision-making bodies

**Table 2:** Recommendations emerged from the roundtable grouped in three categories.

Harmonization of the Current Inconsistencies at National and European Level	Improvement of Management and Organization	Outreach to Relevant Stakeholders
-Standardize technology	-Ensure LB receives adequate expert guidance in terms of interpretation and application	-Raise patient awareness and identify and cooperate with strong patient-advocacy groups
-Standardize clinical approaches	-Require the creation of molecular tumor boards to ensure adequate interpretation of results	-Persuade reimbursement authorities of the need to support molecular diagnostics and LB development
-Standardize pre-analytical practice	-Educate and discuss with colleagues, even where caseloads do not permit individual discussions in real time	-Seek the standardization of national approaches to reimbursement
-Create integrated multicenter-tested workflows covering the requirements for the clinical setting, with SOPs for all phases of laboratory testing, from specimen collection to result interpretation	-Ensure training concerning LB, particularly among pathologists	-Persuade health authorities of the need for the uptake of innovations with molecular diagnostics and LB development
-Set up an international LB standardization alliance	-Create transparency in workflows for clinicians—even with an easy-to-read handbook	-Persuade policymakers to include molecular diagnostics and LB in the national cancer-control plans
-Design and execute adaptive clinical trials based on LB/MRD detection to establish their clinical utility, along with improvements to CTC/ctDNA assays sensitivity and reproducibility	-Train next-generation pathologists who are able to evolve from morphological evaluation to molecular analysis, taking account not just of tissue and cytopathology but also LB and data analysis	-Engage with the European Commission to seek a funding line to support the uptake of LB in the healthcare system
-Set up interventional clinical trials in advanced stages of cancer, with robust and standardized methodologies, along with the development of an algorithm that can combine different circulating biomarkers to obtain a precise tumor profile		-Engage with the public sector and public–private partnerships to support the translation of research and academic work into innovation
-Establish guidelines and SOP for LB for technical variability in the pre-analytical and analytical steps		-Persuade authorities to link diagnostics and therapy more coherently in national regulation and reimbursement, so as to close the gap between predictions based on molecular pathology and access to the drug to treat the mutation detected
-Create a laboratory network that is capable of carrying out the next-generation-sequencing testing of tissue and LB in the context of regional oncological networks		-Maximize the potential of EU actions on cancer and on research to advance the acceptance of and uptake of LB
-Link institutes of research and hospitals to assist in the wider circulation of and access to samples		
-Standardize the preparation of samples and favor consortia such as the European Liquid Biopsy Society (ELBS) in working on protocols		
-Talk about the limitation and uncertainty the LB and patient benefit		
-Ensure recommendations target all types of hospitals across Europe countries		
-Ensure recommendations reach hospitals that do not have much access to testing so that they can at least advise patients about the possibilities elsewhere		
-Ensure integration of the range of information from all sources		
-Explore sending patients with very large mutations to clinical trials across borders		

## Data Availability

Not applicable.
